# On Relaxation of the Symphysis Pubis after Labor

**Published:** 1852-02

**Authors:** 


### OBSTETRICS.

*On Relaxation of the Symphysis Pubis after Labor.*—It is not an uncommon occurrence after delivery, to find relaxation of the pubic ligaments; but from the uncertainty of the symptoms developed by it, it is liable to be mistaken for disease or displacement of the womb. M. Marten, (*Gaz. Médicale,* Juliet,) hopes to throw some light upon the subject.

In all patients suffering from this affection, standing is peculiarly painful and difficult, so that the patient can barely walk a few paces even with the help of crutches. They also complain of more or less dysuria, with frequent micturition. If the hands are placed on the ilei while the patient attempts to walk, she will find that the hip of the leg which rests on the ground is pushed up, while the opposite, from contast seems lower than natural.

In treating this accident, M. Marten makes use of a steel girdle, which entirely encircles the pubis. By means of the support thus afforded, he has known a patient walk with ease, who a few days previously could with difficulty stand upon her feet.—*Prov. Med. Journ.*

---

### RANKING'S ABSTRACT OF THE MEDICAL SCIENCES.

Just as the last form of our Journal was going to press, we received, through the politeness of Messrs. Lindsay & Blakiston, the January number of this most useful periodical, containing a retrospect of the more valuable portions of the journal literature of the last six months. To those acquainted with the merits of this work, we need say nothing in its praise; to all others, we most heartily commend it. Its prompt appearance in its American dress, reflects great credit upon its energetic publishers.

